# Effectiveness of Physical-Activity-Based Interventions Targeting Overweight and Obesity among University Students—A Systematic Review

**DOI:** 10.3390/ijerph19159427

**Published:** 2022-08-01

**Authors:** Julia Pfisterer, Constantin Rausch, Doreen Wohlfarth, Philip Bachert, Darko Jekauc, Kathrin Wunsch

**Affiliations:** Institute of Sports and Sports Science, Karlsruhe Institute of Technology, 76131 Karlsruhe, Germany; julia.pfisterer@student.kit.edu (J.P.); constantin.rausch@student.kit.edu (C.R.); doreen.wohlfarth@student.kit.edu (D.W.); philip.bachert@kit.edu (P.B.); darko.jekauc@kit.edu (D.J.)

**Keywords:** physical-activity-based interventions, overweight, obesity, BMI, change, university students, tertiary education, review

## Abstract

Overweight and obesity, including their prevalence and consequences, reflect a leading public health problem. Studies have already shown that physical activity leads to a reduction in body weight in children and adults. However, the university setting has rarely been investigated. The aim of this review is, therefore, to examine and summarize the effectiveness of physical-activity-based interventions to reduce obesity and overweight in university students. Three databases (PubMed, Scopus, and Web of Science) were searched for relevant studies published in English between January 2010 and February 2022. Quantitative studies conducting a physical-activity-based intervention with overweight or obese university students and reporting changes in BMI were included. Data were described in a narrative synthesis. Out of 16 included studies, 11 reported a significant reduction in BMI. However, all studies except one were able to demonstrate some BMI improvements, whereas all studies reported significant changes in at least one health-related indicator. Aerobic exercises were able to demonstrate the greatest reductions in BMI. This review is the first systematic presentation on the effectiveness of physical-activity-based interventions in overweight and obese university students. Future work should reconsider BMI as the primary outcome if appropriate within the respective study design (i.e., to measure long-term effects). More interventions are needed to improve strategies.

## 1. Introduction

It is already known that physical activity is an essential component of combating overweight and obesity. The positive effects of physical activity on physical and mental health have been demonstrated in numerous studies and reviews [1,2,3]. Nonetheless, in 2016 the WHO identified that 28% of the adults (>18 years) worldwide are not physically active enough. This means that these adults are physically active for less than 150 min per week at a moderate intensity or less than 75 min at a vigorous intensity [4]. Additionally, the rate of overweight and obese individuals increased sharply in recent decades and has developed into a leading public health problem. Being overweight causes cardiovascular diseases, diabetes, musculoskeletal disorders, and different types of cancer [5]. According to the WHO, the prevalence of obesity has almost tripled since 1975. In 2016, 1.9 billion people aged 18 years and older were overweight, of which 650 million individuals were considered obese. Accordingly, in 2016, 39% of the adult population worldwide was overweight and 13% was classified as obese [6]. Depending on the geographical region, the classification of overweight and obese differs; for more details see [6,7]. In addition to the most commonly used method for measuring overweight and obesity, the anthropometric method based on BMI classification, two different approaches can be used to assess overweight and obesity. Indirect measurements of BMI include waist circumference, waist-to-hip ratio, or body fat percentage based on skinfold thickness, whereas direct measurements of body fat percentage include dual-energy X-ray absorptiometry [8]. Even though existing evidence shows that higher education levels seem to be associated with a lower probability of being overweight or obese [9], the prevalence among university students is substantially high [10]. In the ACHA-National College Health Assessment (ACHA-NCHA) III by the American College Health Association (ACHA), 38.1% of undergraduate students were classified as overweight or obese [11]. Peltzer et al. investigated the prevalence of overweight and obesity among 15.746 university students from 22 different countries and concluded that 22% were overweight and obese [10]. Additionally, when compared to the general population, weight gain is five times higher among university students [12]. Especially in the first year of college, university students tend to gain weight, which is referred to as the “Freshman 15” phenomenon. Freshman 15 is the myth that college students gain 15 pounds (6.8 kg) during their first year of university [13]. Vadeboncoeur et al. conducted a meta-analysis of 22 studies on this topic and concluded that 60.9% of students gained on average 7.5 pounds (3.38 kg) of weight during their first year of college [14]. This meta-analysis failed to confirm the myth of the Freshman 15, but weight gain in the first year of university was significant. These numbers are alarming given that university students tend to engage in unhealthy habits such as insufficient physical activity. The ACHA-NCHA survey, for example, demonstrated that less than half of the US college students (42.1%) met the guidelines for active adults in spring 2021, meaning being physically active on at least two days a week at a moderate or vigorous intensity and demanding all major muscle groups [11]. Similar figures have already been observed by Irwin in a review of 35.747 students, stating that half of the students were insufficiently physically active and, consequently, at higher risk for health problems [15]. Furthermore, Keating et al. found in their meta-analysis that around 30 to 50% of college students were not physically active enough to reach any health benefits [16]. Due to the abovementioned reasons, university as a setting should be allocated a significant role in public health promotion.

Considering the high prevalence of overweight and obesity as well as insufficient physical activity among university students and the associated health risks, tailored interventions are needed to address this vulnerable population. The effectiveness of interventions including physical activity on the overweight and obese population has already been investigated in different age groups such as adults and children [17,18,19]. Even though Plotnikoff et al. examined the effectiveness of interventions targeting weight-related behaviors among university students in their meta-analysis [20], these results cannot be generalized to overweight or obese students. Thus, there exists no systematic review which focuses on physical-activity-based interventions for overweight and obese students. In light of findings that increasing physical activity is an important approach to reducing weight and that current reviews and meta-analyses neglect the population of overweight and obese university students, this systematic review aims to investigate the effectiveness of physical-activity-based interventions targeting overweight and obesity among university students.

## 2. Materials and Methods

This study was carried out in accordance with the Preferred Reporting Items for Systematic Reviews and Meta-Analysis (PRISMA) statement guidelines [21].

### 2.1. Eligibility Criteria

Eligible characteristics were based on different items of the PI(C)OC scheme (Population, Intervention, Comparison (optional), Outcomes, and Context) [22]. Overall, selection criteria were kept general at the outset to provide an overview of the topic and to ensure finding all relevant sources. Further specific narrowing was carried out in a later phase.

### 2.2. Types of Participants

Studies that recruited overweight (>25 kg/m^2^) or obese (>30 kg/m^2^) university students in terms of body mass index (BMI) as well as studies where the authors declared that the population was overweight/obese were included. This classification of BMI was used as the WHO expert consultation suggested that researchers should use it to facilitate international comparisons [7]. Other characteristics such as age, gender, socioeconomic, and ethnic characteristics of the students were not further considered or narrowed down.

### 2.3. Types of Intervention

Interventions that were eligible for inclusion had to be conducted in a tertiary educational setting, where university students were recruited to perform a physical-activity-based program. Anthropometric characteristics (height and weight) of the enrolled participants were measured, which could be used for the calculation of the BMI. All interventions encompassing any physical movement were considered for this review. Thus, interventions implementing a fitness, exercise, or sports program were equally taken into account, as were interventions fostering light physical activity, such as walking. Type, duration, guidance, and the number of sessions were not further defined to provide a comprehensive overview of the current study situation. Intervention studies implementing online programs using digital communication or technical devices such as activity trackers to foster physical activity among university students were considered and not further specified in their study design for the abovementioned reason. Interventions without any physical activity component, for instance, focusing solely on diet to target overweight and obesity in university students, were not taken into consideration.

### 2.4. Types of Studies

All quantitative study designs conducting an intervention (including randomized controlled trials (RCT), controlled clinical trials (CCT), cohort analytics, case–control studies, or cohort studies) were included in this review.

### 2.5. Types of Outcome Measures

To measure the change in students’ BMI due to physical activity, an analysis of BMI was the primary outcome measure of the intervention. Changes in other anthropometric characteristics such as body fatness, blood counts, etc. were accepted as additional outcome documentation.

In order to estimate and compare the effectiveness of the interventions, effect sizes were calculated using Psychometrica [23]. The effect size d_ppc2_, according to Morris [24], of studies using an RCT or CCT design in which the control group (CG) did not change their behavior was calculated with formula 3. Formula 4 was used for cohort designs or RCTs and CCTs where the CG changed their behavior. Since none of the studies reported intercorrelations, the effect size d_av_ of Cumming [25] was used, a pragmatic approach often used in meta-analysis.

### 2.6. Information Sources and Search

To examine the effectiveness of physical-activity-based interventions among university students, a structured electronic search of three databases was conducted based on the PRISMA guidelines [21]. PubMed, Scopus, and Web of Science were specified as suitable databases as they comprehensively cover the fields of interest regarding the research question. Only articles published in peer-reviewed journals between January 2010 and February 2022 and written in English were considered for the review. The following keywords and operators were used following the PI(C)OC scheme in all databases. Since the population and the context are congruent, these two terms were merged and searched as follows: students OR university OR college OR tertiary education. Using the NOT operator, some terms relating to the population or context were excluded to restrict the search: adults OR elementary OR children. Interventions were reflected with the keywords as follows: intervention OR treatment OR health promotion OR physical activity* OR exercise OR training OR sport OR fitness. The outcome was searched with the help of: overweight OR obesity OR BMI. The search terms had to be adapted regarding title, abstract, and full-text search for the specific databases. The appendix contains the complete and final search terms of all three databases. In addition, authors were contacted via ResearchGate to obtain eligible studies that were not publicly available. Furthermore, while processing the data extraction, the bibliography of each study was searched for additional potentially relevant studies. Searches began on the first of December 2020 and ended with the last search query in March 2022.

### 2.7. Study Selection

A pre-selection of studies was conducted by each author individually through title screening, saving those which included relevant keywords of inclusion criteria in Citavi 6.11 (Swiss Academic Software GmbH, Wädenswil, Switzerland) to avoid duplications. In the next step, all authors first performed abstract screenings of an equitable number of the pre-selected studies individually to check for inclusion and exclusion characteristics. Secondly, each abstract was again discussed by all authors together to avoid omitting relevant studies. As a consequence, studies that did not have an intervention design, did not target a university students’ population, and did not report BMI results were removed. In the last step, studies were checked for inclusion and exclusion criteria more thoroughly by full-text analysis with triple approval to reduce bias.

### 2.8. Data Collection and Data Details

Data extraction was performed in a standardized manner, with each author examining an equal number of studies individually and extracting all relevant information from the respective studies into spreadsheet format. Based on the results of data extraction, the authors discussed ambiguities and jointly decided which studies should ultimately be included for analysis in the review.

### 2.9. Quality Assessment

Quality assessment for the selected studies was conducted by using the EPHPP Quality Assessment Tool for Quantitative Studies (Effective Public Healthcare Panacea Project, [26]). This assessment tool is an elaborated instrument for evaluating the quality of a study and can be used in a wide range of health-related topics to develop recommendations for study findings. It includes several items helping to classify a study according to its quality from one (strong) to three (weak). Each rating is described for: (1) selection bias, (2) design, (3) confounders, (4) blinding, (5) data collection methods, and (6) withdrawal and dropouts [26].

Every author rated each full text individually at first. Results of the individual ratings were then discussed by all authors together and, in case of any ambiguities, a joint decision for a final rating was made [27]. Full-text analysis, data extraction, and quality assessment were completed by April 2022.

### 2.10. Sythesis of Results

The synthesis of results was based on the Guidance on the Conduct of Narrative Synthesis in Systematic Reviews [27]. This approach by Popay et al. uses a general framework to describe the four elements of the narrative synthesis process, which are presented as follows: developing a theoretical model, preliminary synthesis, exploring relationships within and between studies, and assessing the robustness of synthesis.

Following Plotnikoff et al. [28], it was analyzed whether the selected studies based their intervention on a theoretical model, focusing especially on social cognitive theories as they can be used to explain health-related behavior, including physical activity [29]. To develop a preliminary synthesis, the authors identified similarities and differences among study findings, partially using charts and tables. In the process of analyzing the results of each study, the authors yielded, with the help of their methods, further new insights. In the next step, these preliminary results were then discussed in detail and compiled in group sessions. Based on the direction and magnitude of the effects, associations and patterns between the interventions were identified, which needed to be explained in the third step. To this end, the authors analyzed heterogeneity across studies, such as variability in outcomes, study designs, study populations, interventions, and settings. This step of the analysis is characterized by the relationship of outcomes within and between studies. Finally, each outcome of the included studies was evaluated to be able to assess the strength of the synthesis product. As already described before, the results of the quality assessment were considered and could contribute to the overall assessment of the review results. With the help of the quality assessment, it could be identified if results could be generalized and transferred to other populations or contexts.

## 3. Results

A total of 3231 hits were obtained in the PubMed, Scopus, and Web of Science databases using the previously defined search terms. Individual screening per database for matching keywords in the title and abstract led to a selection of 93 studies. The exclusion of studies based on the selection criteria that did not include an intervention design, student population, or physical activity ultimately led to a large reduction in eligible studies.

**Figure 1:**
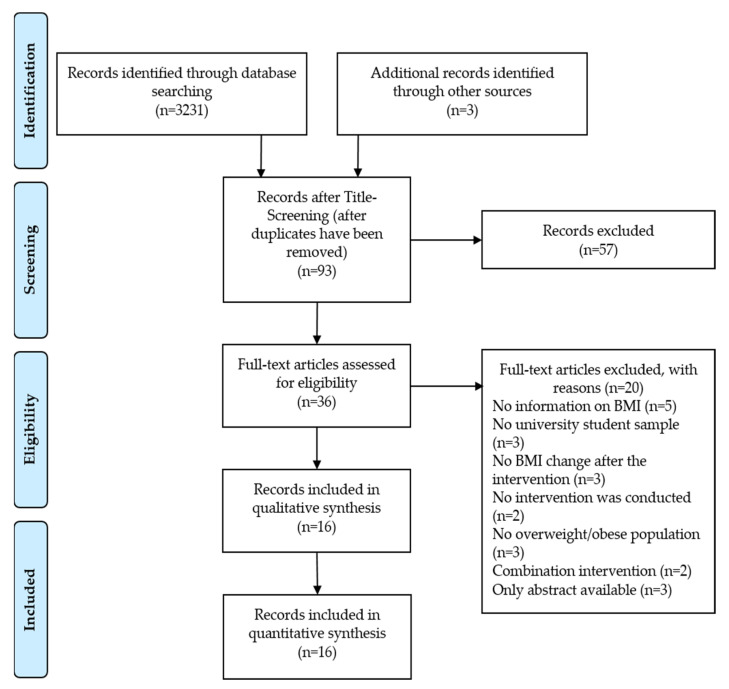
Flow chart of study selection process.

By using the literature management program Citavi 6.11 (Swiss Academic Software GmbH, Wädenswil, Switzerland), duplicates were immediately removed, leaving 93 studies that were subjected to a more detailed abstract screening. In particular, studies were excluded that did not correspond to an intervention or did not examine a student sample. Web-based interventions, i.e., interventions that promote physical activity via online communication, were equally shortlisted, resulting in 36 studies after the abstract screening. In some cases, no information was available on the BMI of the group after the intervention, as the focus of the studies was on other dependent variables; therefore, 15 studies were excluded. Thus, 21 studies were still available for the full-text analysis. Of these, two additional studies were identified through the full-text analysis as using online communication to motivate physical activity, but not conducting an actual training program. Full-text access was not available for three other studies. As no feedback was received after contacting the authors of these three studies via ResearchGate, none of them could be included. The abovementioned five studies were therefore excluded, resulting in 16 studies that met the specified inclusion criteria. The forward and backward search of the bibliography via the website Connected Papers did not yield any additional sources. Figure 1 shows the literature search process.

### 3.1. Study Characteristics

#### 3.1.1. Methods

The 16 studies that were ultimately included in the review were all published in English between 2012 and 2021. The interventions differed in their study design with seven using CCTs [30,31,32,33,34,35,36], four using RCTs [37,38,39,40], and five using cohort studies with a single group and pre–post design [41,42,43,44,45]. The interventions were conducted in different countries around the world, including four in the USA [31,40,41,42], four in China [34,35,37,39], two in Korea [33,43], and one each in the United Emirates [30], Indonesia [32], South Africa [44], Iran [38], Jamaica [45], and in Saudi Arabia [36].

#### 3.1.2. Subjects

A total of 969 students were included in the final analyses. Sample sizes varied from 10 to 300 participants, with only 3 of the 16 studies having a sample size of at least 100 [31,34,39]. Only DiFrancisco-Donoghue et al. [31] ensured an equal gender distribution in their sample. Chen et al. [37], Pacholek et al. [36], and Sun et al. [39] studied only male subjects, whereas the other interventions involved either exclusively female or predominantly female subjects. In eight studies, the main inclusion criterion was a BMI of at least over 25 [31,32,37,38,39,41,42,44]. Ha and So [33] and Zhang, W. and Yu, L. [35] used a body fat percentage of more than 30%, whereas Siqiang [34] used a body weight of more than 58 kg to declare the sample as overweight. Winters-Stone et al. [40] recruited students who were overweight or were at risk of being obese (BMI > 22 kg/m^2^), and in the remaining studies, being overweight was not an inclusion criterion. Nonetheless, Roopchand-Martin et al. [45] used a BMI above 25 to divide the sample into an overweight and a normal weight group for analysis in addition to the total group. Pacholek et al. [36] divided the sample into two overweight groups to analyze the change in overweight students based on the two different exercise programs. Dalibalta et al. [30], in turn, used the same group allocation to use the overweight group as the intervention group (IG) and the normal weight group as the CG. Lee et al. [43] did not describe their main inclusion criteria in detail. Nonetheless, the sample was declared inactive and had, in total, a BMI over 25 kg/m^2^ (25.14 kg/m^2^).

#### 3.1.3. Interventions

Subjects in all interventions received a physical-activity-based program that varied in type, intensity, and frequency. The training sessions for the IGs included aerobic training [34,41,42], a combination of aerobic and resistance training [35,36], the combination of strength and aerobic training [33], high-intensity interval training [32,38,39], high-intensity circuit training [43], a specific exercise or sport, namely, dancing, Tae Bo, whole-body vibration (WBV), or volleyball [36,40,44,45], or an unspecified training program [30,31]. It should be noted that the studies by Gifari et al. [32] and Zhang, W. and Yu, L. [35] included an additional nutrition program in their IGs; however, these were analyzed separately. Chen et al. [37] carried out a cardio-based cycling program. The CGs mostly received either a different exercise program [30,37,39] or instructions to maintain their usual activity level and eating behavior [33,35,38,40]. In half of all included interventions, exercise was provided three times a week [30,32,33,35,38,40,43,44], whereas in the other interventions sessions ranged from two to six times a week. The majority of the interventions were limited to moderate intensity or a heart rate between 50% and 85% of maximum heart rate [30,32,33,34,35,38,39,41,42,43,44,45]. Only one intervention had participants working at a predetermined low intensity of 40% VO_2max_ [37] and one was based on a subjective feeling with maximal effort [36]. Out of the 16 interventions, 4 successively increased the load by either increasing the training duration [45] or the intensity of the training sessions after a certain number of weeks [40,43,44]. The intervention duration varied between studies from 4 weeks [36,43] to 39 weeks [31]. The most common intervention duration was twelve weeks [33,34,37,39,41,42], followed by eight weeks [30,32,38].

#### 3.1.4. Outcomes

All included studies provided information on the change in BMI after the intervention and, for this purpose, collected the anthropometric parameters of the subjects. These included body weight and height for the calculation of BMI. In addition, all studies also examined other dependent variables such as body composition, blood pressure, blood lipid levels, or behavioral factors. Thus, BMI was not the sole factor of interest in any study. However, for this review, the necessary information on the change in BMI could be extracted from all studies. Study characteristics are summarized in detail in Table A1.

### 3.2. Risk of Bias within the Studies

Five studies were rated as strong using the global rating from the EPHPP tool [31,33,38,39,40], whereas seven received a moderate rating [32,34,35,37,41,42,44] and four received weak ratings [30,36,43,45]. For the selection bias category, only Gifari et al. [32] received a strong rating as they described the selection of their sample as randomized. Even though the studies by Pacholek et al. [36] and Zhang, W. and Yu, L. [35] declared a randomization process in their study population, they had a homogenous sample in terms of sex. In addition, the remaining 13 studies did not explicitly state a randomization process. Furthermore, Roopchand-Martin et al. [45] did not report how individuals were selected and what percentage of selected individuals agreed to participate and were thus assigned a weak rating. Therefore, all studies, except the study by Roopchand-Martin et al. [45] and Gifari et al. [32], received a moderate rating. In sum, 11 out of 16 studies did not describe what percentage of the selected individuals ultimately gave their consent to participate in the intervention [30,31,33,34,35,36,38,41,42,43,45]. Strong ratings were predominantly assigned to the study design, as eleven of the included studies were either RCTs [37,38,39,40] or CCTs [30,31,32,33,34,35,36], which should receive the best rating according to the specifications of the assessment tool. The five cohort studies without CGs [41,42,43,44,45] were thus rated as moderate. Information on confounders differed greatly between the studies so that half of the 16 included studies were given the highest rating, whereas the other half received the lowest. The highest ratings were allocated when there were no crucial differences between groups before the intervention [31,33,34,35,37,38,39,40]. The weak ratings were assigned when: (1) group differences existed before the intervention and it was not described if relevant confounders were controlled [30], (2) there was only one group due to the study design [41,42,43,44,45], or (3) control of confounders was not described [32,36]. For blinding, Chen et al. [37] and Lee et al. [43] received a weak rating, since either the awareness of the outcome assessors was not described or the participants and/or assessors were aware of the exposure. The remaining 14 studies received a moderate overall rating on blinding, as none but one study described the blinding process. Only Winters-Stone et al. [40] provided information on the blinding of test administrators and subjects, but stated that the research question was communicated to the subjects; thus, only a moderate ranking was assigned here as well. BMI was collected in all studies using the standardized methods of weight recording and measuring height. Consequently, the methods of data collection were all rated as strong. Withdrawals and drop-outs were described in detail in 8 out of 16 studies, with reasons and/or numbers given [31,33,37,38,40,42,44,45]. Moreover, 11 of the 16 studies had a follow-up rate of 80% or greater [31,32,33,37,38,39,40,41,42,44,45], thus strong ratings were assigned here. In addition, 5 of the 16 studies [30,34,35,36,43] did not provide any information in this regard and, thus, received a weak ranking. Table 1 shows the global rating as well as its composition for each study.

### 3.3. Results of the Individual Studies

Out of the 16 included studies, 11 reported a significant reduction in BMI within IGs after the intervention at a predetermined significance level of five percent [30,32,34,35,37,38,39,40,41,43,44]. Even though the studies by DiFrancisco-Donoghue et al. [31]; Ha and So [33]; Joseph et al. [42]; Pacholek et al. [36]; and Roopchand-Martin et al. [45] did not produce significant BMI reductions, they showed some decrease in BMI ranging from −0.2 to −0.9 as well. The IG of the study by Roopchand-Martin [45] had the smallest decrease of −0.2, whereas Joseph et al. [42] had the highest non-significant reduction in BMI of −0.9. It should be noted that DiFrancisco-Donoghue et al. [31] showed a BMI reduction only for female participants of the Fitbit-Plus group (−0.5) and the male Fitbit-Only group (−0.2), but did not report any *p*-values and, therefore, no conclusion can be drawn on the significance. Pacholek et al. [36] reported a reduction of −0.3 in BMI for the volleyball IG. Finally, Ha and So [33] showed a slight decrease in BMI in the IG of −0.21.

Of the eleven included studies which used a RCT or CCT design, eight reported significant results in terms of changes in BMI [30,32,34,35,37,38,39,40]. However, since the CGs in five studies using a RCT or CCT design changed their behavior as well, only four studies could be taken into account for a comparison between the IG and CG [33,35,38,40]. Here, three of four studies found significant differences between the CG and IG [35,38,40]. In the five studies in which the CGs changed their behavior [30,31,34,37,39], three studies reported significant BMI reductions in the IG and CG [30,34,39]. Chen et al. [37] only reported significant reductions in the IG but not in the CG. DiFrancisco-Donoghue et al. [31] gave insufficient information on significance and just reported the total differences in body composition for the overweight population. The studies by Gifari et al. [32] and Pacholek et al. [36] were declared as CCTs but did not include a CG in their design. Like the abovementioned studies, where the CGs changed their behavior, these studies used three [32] or two [36] IGs to compare the effectiveness of the intervention. Pacholek et al. [36] found a non-significant reduction in BMI in both groups, whereas in the study by Gifari et al. [32], one group significantly decreased their BMI.

In four of the seven included CCTs significant improvements in BMI were reported [30,32,34,35]. Dalibalta et al. [30] conducted the same exercise program with both the IGs (*n* = 14, MBMI = 28.7, SD = 3.27) and the CG (*n* = 32, MBMI = 21.6, SD = 2.05). Groups differed regarding the average BMI, thus there was an overweight IG with a BMI of at least 25 and a normal weight CG with a BMI of less than 25. Both groups received an exercise training program that was not described in more detail. After eight weeks, BMI had decreased significantly in both the CG (MBMI = 21.1, SD = 2.00, d_av_ = 0.247) and the IG (MBMI = 28.1, SD = 2.79, d_av_ = 0.198). However, BMI was reduced by a greater extent in the normal weight group (2.31%) than in the overweight group (2.09%). Siqiang [34] chose aerobic exercise for the IG (*n* = 50, MBMI = 25.5, SD = 2.70), whereas the CG (*n* = 50, MBMI = 26.8, SD =3.40) only had to take calcium pyruvate twice a day. After twelve weeks of intervention, there was a great significant reduction in BMI in the overweight CG (MBMI = 24.1, SD = 2.90, d_av_ = 0.857) and an even greater reduction in the IG (MBMI = 21.6, SD = 3.10, d_av_ = 1.345). Zhang, W. and Yu, L. [35] combined aerobic exercises and resistance training for their IG (*n* = 20, MBMI = 22.06, SD = 0.98) and asked the CG (*n* = 20, MBMI = 22.54, SD = 0.71) to maintain their behavior. After 16 weeks, BMI within the IG was significantly reduced by 1.9% (MBMI = 21.64 SD = 0.85, d_ppc2_ = 0.641), whereas the CG showed an increase in BMI of 0.62% (MBMI = 22.68, SD = 1.11). Gifari et al. [32] combined HIIT exercises with pre-meal water intake (PWI) for one of their IGs (*n* = 9, MBMI = 26.6, SD = 3.6) and exclusively HIIT exercises for the other IG (MBMI = 25.9, SD = 2.4). The HIIT + PWI group significantly reduced its BMI by 1.95% (MBMI = 25.2, SD = 3.2, d_av_ = 0.152), whereas the exclusively HIIT group failed to significantly reduce the BMI with a decrease of 0.38% (MBMI = 25.8, SD = 2.4, d_av_ = 0.042). Small, non-significant decreases can be seen for the IGs of DiFrancisco-Donoghue et al. [31], Pacholek et al. [36], and Ha and So [33].

All four included RCTs that reported significant results [37,38,39,40]. Chen et al. [37] used a combination of low-intensity cycling and blood flow restriction training (BFRT) by wearing air pressure belts for their IG (*n* = 18, MBMI = 30.10, SD = 0.95). The IG was compared with a CG (*n* = 19, MBMI = 30.30, SD = 1.08) that also used low-intensity cycling but did not perform BFRT. After the intervention period of twelve weeks, BMI was reduced within both groups. The IG was able to significantly reduce its BMI by 3.65% (MBMI = 29.0, SD = 1.79, d_av_ = 0.803), whereas the BMI in the CG was non-significantly reduced by 1.32% (MBMI = 29.90, SD = 1.44, d_av_ = 0.317). Moravveji et al. [38] used the principle of successive weekly increases in training units. A 1200 m run was completed three times per week and increased by 400 m each week. Subjects were divided into: (1) a continuous group (*n* = 10, MBMI = 26.77, SD = 1.53), completing the 1200 m without a break, (2) an interval group (*n* = 12, MBMI = 27.57, SD = 1.02), which ran 400 m three times with a two-minute break of walking between runs, and (3) a CG (*n* = 9, MBMI = 27.57, SD = 1.62). The CG was asked to maintain its normal behavior and was instructed not to participate in any other training program during the intervention period. After eight weeks, BMI significantly decreased in both the continuous group (MBMI = 26.08, SD = 1.21, d_ppc2_ = 0.704) and the interval group (MBMI = 26.82, SD = 0.98, d_ppc2_ = 0.896). The CG, on the other hand, experienced a slight increase in BMI of 1.70% (MBMI = 28.04, SD = 1.46). Sun et al. [39] performed high-intensity interval training with the IG (*n* = 150, MBMI = 30.85, SD = 3.79) and aerobic training with the CG (*n* = 150, MBMI = 30.54, SD = 3.86). After twelve weeks of the intervention, there was a significant reduction in BMI within the IG (MBMI = 28.17, SD = 2.89, d_av_ = 0.802) and the CG (MBMI = 28.45, SD = 2.76, d_av_ = 0.631). The RCT by Winters-Stone et al. [40] differed somewhat from the other studies in its training program. Training sessions in the IG were performed using a whole-body vibration method (*n* = 37, MBMI = 28.24, 95% CI [23.87; 32.60]). The degree of vibration gradually increased in the first six weeks and was adjusted to 50 Hz in weeks 7–24. The CG (*n* = 40, MBMI = 28.27, 95% CI [26.48; 30.07]) was expected to maintain its usual eating behavior and physical activity level throughout the intervention period. Even though a slight increase in BMI of 0.22 (95% CI [−0.59; 1.03]) was found at a non-significant level in the IG, when analyzing only participants with high adherence (MBMI = 27.02, 95% CI [22.34; 31.71]) a small significant reduction of 0.04% could be found. As Winters-Stones et al. [40] provided insufficient information, no effect size could be calculated.

Three of the five that included cohort studies declared a significant reduction in BMI [41,43,44]. The subjects in the study of Joseph et al. [41] (*n* = 15, MBMI = 33.27, SD = 6.53) participated in aerobic training in the form of walking and were also given the choice between exercising by themselves and cardiovascular group training. After the intervention of twelve weeks, the average BMI was reduced by 2.10% (MBMI = 32.57, SD = 6.62, d_av_ = 0.106). Lee et al. [43] conducted high-intensity circuit training (*n* = 10, MBMI = 25.14, SD = 2.51) for four weeks, three times per week, and the BMI of their intervention population was reduced by 3.58% (MBMI = 24.24, SD = 2.60, d_av_ = 0.352). The training sessions of Mathunjwa et al. [44] consisted of Tae Bo lessons in which subjects participated three times a week for ten weeks. After five weeks of moderate intensity, training was increased to high intensity in weeks six to ten. The BMI of the group (*n* = 60, MBMI = 32.26, SD = 5.65) had decreased by 6.66% (MBMI = 30.11, SD = 5.46, d = 0.2). Joseph et al. [42] used an Internet-enhanced approach combined with real-life physical activity interventions for twelve weeks at a moderate level (*n* = 25, MBMI = 33.80, SD = 5.70). Even though results were not significant, a decrease in BMI was shown after the intervention (MBMI = 32.90, SD = 6.00, d_av_ = 0.154). Roopchand-Martin et al. [45] conducted a dance training on the XBOX Kinect using the program Just Dance. During the six-week intervention, dance duration was increased by fifteen min every two weeks, whereas the frequency was reduced by one session per week to maintain participation rates. After six weeks of moderate training the BMI of the subjects (*n* = 24, MBMI = 30.51, SD = 5.18) decreased at a non-significant level by 0.66% (MBMI = 30.31, SD = 5.39, d_av_ = 0.038). Results of the included studies can be seen in more detail in Table A2.

### 3.4. Synthesis of Results

Behavioral interventions are characterized by their complexity and variability. Therefore, the included studies are distinguished by considerable heterogeneity in terms of methods, participants, intervention approaches, and other characteristics. As mentioned by Popay et al. [27], a primary reason for choosing a narrative approach is to investigate the differences in the included studies. Physical-activity-based interventions vary substantially across their intervention approaches. Thus, the focus of this systematic review is the presentation and description of the study characteristics, quality, and outcomes, as well as a qualitative analysis of the included studies.

Theory-based interventions have been shown to have a positive impact on physical activity for adolescents [28]. Of the studies included in this review, only the study by Joseph et al. [42] was theory-driven and based its intervention on the social cognitive theory. Since only one study used a theoretical framework, no conclusion can be drawn on whether the results of the individual studies can be attributed to the theoretical background. Likewise, as only two of the included studies used a web-based approach and did not yield any significant results [31,42], no conclusion can be drawn about the effectiveness of technological devices or web-based approaches.

In all studies that included a CG in their study design, excluding DiFrancisco-Donoghue et al. [31] and Dalibalta et al. [30], the percentage of reduction in BMI was always higher in the IG. Although not all studies showed significant effects on BMI, all included interventions that reported at least one significantly improved health-related indicator. Those health-related indicators included improvements in body fat percentage, step counts, sedentary behavior, maximal oxygen consumption, resting heart rate, or serum biomarkers.

However, it is noticeable that all studies, except Winter-Stone et al.’s [40], showed a decrease in BMI in at least one of their IGs, suggesting a tendency toward weight loss. Of note, although Winter-Stone et al. [40] found no BMI reductions when all participants in the IG were included in the analysis, when controlling for high adherence of at least 80% of intervention sessions, there was a significant improvement. This may indicate that high participant adherence may be an important factor in the success of an intervention.

Furthermore, the study by Gifari et al. [32] produced significant BMI improvements of 1.95% in their combination group, which included pre-meal water intake and high-intensity training. Considering that the effect was lower when conducting only HIIT training (−0.38%), and there was no reduction in the group consuming only water before meals (0.38%), the combination of both approaches could positively influence the intervention effects.

The largest percentage reductions in BMI in the IGs were achieved in the studies by Siqiang [34], Sun et al. [39], and Mathunjwa et al. [44], with a decrease of 15.29%, 8.69%, and 6.66%, respectively. In comparison to the other studies, except Winters-Stone et al.’s [40] and Zhang, W. and Yu, L. [35], these interventions had a relatively large sample size, ranging from 60 to 300, with all participants being overweight. Conversely, the four studies which reported *p*-values and did not produce any significant reductions in BMI had a sample size smaller than 29 [33,36,42,45]. This small sample size may have resulted in the studies being underpowered and, therefore, not showing significant improvements.

The four studies that reached the largest BMI reductions [34,37,39,44] conducted only aerobic exercises. Since aerobic exercises tend to have a higher energy expenditure than resistance training, it is considered to be more effective in reducing body weight and fat mass [46] and could therefore explain the positive impacts on BMI changes. Conversely, the study by Ha and So [33], for example, implemented resistance training in their intervention, resulting in a decreased body fat percentage and increased muscle mass, which leads to small changes in body weight and, thus, no significant improvements in BMI could be seen. No gender differences could be detected, since two of the samples of the four studies were exclusively male and two exclusively female. Even though it could be suggested that aerobic exercise is the most effective method for a BMI reduction for both men and women, representative samples concerning gender would be necessary to conduct gender-specific analyses.

It is noticeable that only one of the included studies reported effect sizes [44]. It was possible to calculate effect sizes for another 13 studies, which should be seen as approximate values. Half of the 14 studies did not reach the recommended threshold by Cohen [47] for a small effect of 0.3 [30,32,33,36,41,42,45]. Furthermore, although Mathunjwa [44] did reach one of the largest percentages in BMI reductions, only a small effect can be reported here. One explanation could be the rather large variance of BMI in the sample before as well as after the intervention. For Siqiang [34] and Sun et al. [39], on the other hand, the calculated effect sizes show a strong effect and, thus, appear to be consistent with the reported high percentage of BMI reduction. Here, the standard deviation was substantially lower in contrast to the study by Mathunjwa [44]. Interestingly, although the highest BMI reductions do not necessarily coincide with the strongest effects, the highest effect sizes are also evident in those studies that implemented interventions involving aerobic exercise [34,37,38,39].

Taking the study quality into account, only three of eleven studies with significant results received a strong global rating [38,39,40], six a moderate global rating [32,34,35,37,41,44], and two a weak global rating [30,43]. This leads to the assumption that the results shown above should be interpreted cautiously and should be considered when generalizing the results. Since the included studies provided highly homogenous samples, only one strong rating for selection bias could be assigned [32]; therefore, generalization of the results to different populations should be carried out with caution. Significant BMI reductions were achieved in five of the six studies with a strong rating in study design and that used solely aerobic exercise in their intervention [33,35,38,39,40]. Nevertheless, no clear patterns regarding study design, intervention duration, frequency, and intensity can be identified in the totality of the included studies.

## 4. Discussion

The current review identified 16 studies that examined the effects of physical-activity-based intervention programs to reduce BMI in students within the tertiary sector. Although only eleven studies declared significant reductions in BMI, all studies except one showed at least some reduction in BMI, indicating a trend toward the effectiveness of the intervention programs. In the majority of the studies, BMI was not the primary outcome. Therefore, the effectiveness of the intervention is additionally reinforced by the fact that at least one health-related indicator was significantly improved in each study (e.g., body fat percentage, weight loss, lipid levels, resting heart rate, maximal oxygen uptake, less sedentary behavior, or a higher step count). The greatest improvements in BMI were achieved in studies that performed exclusively aerobic exercises in their intervention and had a relatively large sample size (N > 60). This can also be supported by considering the effect sizes, with the strongest effects observed in studies involving aerobic exercise.

No clear pattern regarding significant BMI reductions could be derived from study design. Additionally, no distinct trends in duration, frequency, and intensity of the intervention programs could be observed because of substantial heterogeneity among studies showing significant improvements in BMI. Since only three studies with significant results received a strong global rating and most of the studies had a highly homogenous sample, which consisted mostly of female participants, the results should be interpreted cautiously, and global generalizability should be questioned. To account for innovative developments in intervention opportunities, studies in which no traditional training sessions were conducted were explicitly included. In these studies, attempts were made to promote the participants’ physical activity with the use of websites and/or messages. However, the two included web-based interventions failed to report significant results in terms of BMI.

Between 1980 and 2008, the prevalence of obesity nearly doubled [6]. Overweight and obesity is a current topic in all different age groups. To avoid an even more overweight and obese population, it is important to act now. There are already many approaches to increase physical activity and reduce BMI. Positive effects of such interventions have been confirmed in adults and children [2,48,49,50,51,52,53,54]. Long-term findings on physical activity were highlighted by Reiner et al. [2] in their review, which included overweight and obesity in adolescents and adults: Over five years, weight change was examined concerning physical activity. It was found that those who reduced their daily physical activity gained considerable weight. Individuals who maintained their activity did not gain weight and, in turn, individuals who increased activity lost weight. Hankinson et al. [49] studied the change in BMI of adults older than 20 years with high habitual activity over 20 years. They found a smaller increase in BMI, waist circumference, and weight per year compared to subjects with low habitual activity. Furthermore, maintaining a higher level of activity in adulthood was able to reduce the subjects’ weight gain over their lifetime. Morano et al. [53] also found improved physical activity with a simultaneous decrease in BMI in children. The review by Brown et al. [50] showed that children aged 6 to 12 years and adolescents up to 18 years have a lower risk of obesity from physical activity alone. By adding physical activity, they could show a low to moderate reduction in BMI from 6 to 18 years of age [50]. In summary, the results of these studies and reviews suggest that higher levels of physical activity lead to lower weight gain or reduce the BMI of the subjects. This systematic review confirms that physical activity can help to reduce BMI in obese and overweight students. Although not all studies of this systematic review show significant results, it can be concluded that physical activity can improve BMI in overweight and obese students in tertiary institutions. Accordingly, the systematic review can be integrated into the current state of research, as 11 out of 16 studies were able to demonstrate a significant reduction in BMI among overweight and obese students and only one study was unable to reduce the BMI of the participants through physical activity. Nonetheless, the prevalence of physical inactivity has risen sharply in recent years [55], even in early childhood and adolescence [54]. As already mentioned, there are numerous interventions designed to counteract the risk factors of physical inactivity, such as excessive body fat percentage, overweight and obesity, unhealthy lipid profile, high blood pressure, etc., through physical activity. All these risk factors could result in cardiovascular diseases, strokes, etc., or even in an increased morbidity [55,56]. Since physical inactivity is especially prevalent among university students, interventions designed to mitigate these risk factors are necessary.

Interventions and reviews have already demonstrated that the above-mentioned risk factors could be significantly reduced through physical activity both in children and adults [50,51,53,54]. In addition to BMI, the physical-activity-based interventions in this systematic review have shown further improvements in other risk factors of physical inactivity. In line with the current literature, this systematic review could therefore demonstrate that physical activity is useful in reducing or even eliminating the risk factors associated with physical inactivity. Relaxation techniques should be used as an additional tool to implement the approach of comprehensive health promotion. Moore and Cunningham [57] stated in their systematic review that exposure to higher stress was associated with poorer nutrition and, additionally, higher body weight. Conversely, a higher level of stress could be an obstacle to weight loss. In addition, Stults-Kolehmainen and Sinha [58] were able to demonstrate in their systematic review that objective stress (e.g., life events), as well as subjective stress (e.g., distress), impair efforts to be physically active. On the other hand, physical activity is known to be stress-buffering, as regular physical activity can buffer the negative consequences of stress on health, which is postulated by the stress-buffering hypothesis [59,60]. Given that the target population of this systematic review was university students who are particularly exposed to high levels of stress [61,62], relaxation strategies should be considered for a comprehensive treatment of the increasing prevalence of overweight and obesity and physical inactivity.

At the level of the individual studies used for this review, some limitations can be identified. Particularly, the insufficient and homogeneous sample size is frequently cited, which may impair the informative value of the results and make generalization to the general population difficult. Another limitation arises from the inadequate control over the intensity in some studies. Therefore, all studies which did not control the intensity with objective indicators (e.g., heart rate) impair generalizability in terms of highest effectiveness according to the intensity. This issue could be additionally exacerbated by the varying adherence to the training program. Even though eleven of the included studies showed significant improvements after the intervention, it should be considered that the intervention periods of these studies lasted only up to four months. Therefore, it is not possible to determine any long-term effects of the conducted interventions. Concerning statistical analysis, only one of the included studies reported effect sizes in addition to the significance level [44]. Since *p*-values always refer to the sample size in contrast to effect sizes [47], effect sizes should be reported more frequently to facilitate detecting patterns across studies and to infer the effectiveness of the included studies. Even though effect sizes could be computed with Psychometrica for the remaining studies, they can only be seen as approximate values.

At the review level, it is important to note that the heterogeneity of the included studies in terms of sample size, gender, type and duration of physical activity, duration of the intervention, and BMI of subjects aggravated to identify patterns across the studies. As the included interventions were conducted in countries with different cultural backgrounds, a cross-cultural effect could be suspected. However, considering the low number of studies and the wide range of existing cultural practices, beliefs, and behaviors, the findings of this review cannot be generalized. They can only serve as a guide for further investigation of effective strategies to address overweight and obesity for a given cultural setting. Additionally, only 31% of the included studies were of strong quality, thus generalization should be taken with caution. Since there was no possibility to translate studies that were in a language other than English or German, the search was limited to the English language from the beginning. In addition, limited access to the full text of nine potentially suitable studies made it difficult to select appropriate studies. Due to these organizational limitations, there is a possibility that other relevant research from the literature could not be included in this review. The choice of BMI as the dependent variable of the intervention proved to be the biggest limitation and bias. Frequently, studies had to be excluded because they reported on body characteristics other than BMI, such as body fat levels. As the relationship between BMI and body fat mass is nonlinear, individuals having the same BMI may have different body compositions (e.g., varying body fat percentages or muscle mass) [63]. Thus, with regard to the effectiveness of physical activity, the main concern is whether a reduction in BMI is associated with a reduced fat mass at all, or conversely, especially for novice exercisers, whether an increase in muscle mass does not initially translate into an increased BMI.

The present review was deliberately limited to the aspect of physical activity. However, since a promising feature of the intervention studies by Brown et al. [50] and Martin et al. [54] that examined children and adolescents is the implementation of a program combining physical activity and dietary changes, future reviews should additionally include the aspect of nutrition. In addition, there is a need to further investigate the effectiveness of aerobic exercises in overweight and obese university students, as well as identifying which types of aerobic exercises are most effective. More intervention studies tailored to the distinct cultural backgrounds in this field are needed to detect cross-cultural patterns. As adherence can play an important role in the success of an intervention, it is important to develop interventions in which participants are more likely to participate regularly. In this regard, it might be useful to differentiate different levels of adherence to gain more comprehensive data, which helps to identify patterns of low and high adherence. This, in turn, would serve as a basis for removing barriers for participants in future interventions and for developing recommendations for practitioners. Since another study demonstrated significant BMI reductions by combining pre-meal water intake with HIIT training sessions, the role of water consumption in combination with exercising represents a promising approach to weight loss that needs further investigation, especially regarding different types of exercises. Furthermore, as the BMI is prone to bias, it should be reconsidered as an appropriate variable to capture the change in body characteristics of a physical activity intervention for future reviews. For this reason, it is important that future research adopts a comprehensive health perspective by examining other health-relevant indicators. Additionally, future research should ensure larger sample sizes as well as representativeness of the sample to facilitate generalization. Since Bothmer and Fridlund [64] concluded that male students are less interested in health enhancing activities, comparative analyses by gender can also help to better understand gender-specific needs of overweight and obese university students. In order to design tailored interventions and programs, studies should ensure an equal gender distribution. To be able to assess long-term effects, longer intervention periods need to be conducted. Combining different training programs for future research might also be a promising approach for reaching better effects. Similarly, as university students are especially exposed to high levels of stress which, in turn, is known to impede weight loss, relaxation strategies should be considered in future interventions. Web-based approaches, using, for example, messaging or informative websites, and technical devices, such as activity trackers, are promising approaches to foster physical activity. They can not only facilitate sampling and flexibility as an organizational aspect, but also help to supervise the intensity of physical activity during the implementation. To be able to set optimal stimuli for participants, more research and interventions with a study design based on a theoretical approach are needed to identify key concepts and to be able to better understand the mechanisms of change behind the intervention effects. In general, more intervention studies of physical activity in overweight and obese university students are needed to gain insight into optimal types of interventions, their frequency, and their duration to explore more contemporary and sustainable opportunities.

As the highest BMI reductions were achieved in interventions conducting aerobic exercises, it might be important for practitioners to focus on exercises promoting aerobic energy expenditure to achieve BMI improvements. Even though this needs to be approved by further research, this method can still serve as a guide. Given that one study demonstrated that high adherence was crucial for significant BMI reduction, methods to ensure consistent participation might be critical for the effectiveness of an intervention and should be considered by practitioners. The present review provides policymakers with a more robust evidence base because it is, to the best of the authors’ knowledge, the first systematic review that assessed the effectiveness of physical-activity-based interventions in overweight and obese university students. It showed that such interventions positively impact the health of overweight and obese students and can help them reduce weight. Policymakers should, therefore, encourage developing and implementing physical-activity-based programs designed for overweight and obese university students. Embedding such programs within the university facilities can facilitate access for the students and account for the cost-effectiveness, which is often a major issue in health promotion programs. The university as a setting offers highly educated staff coming from a variety of health disciplines, a learning environment, and a large number of students who are still developing their lifestyle skills and behavior. Health promotion programs within the universities can thus be an effective tool for short- and long-term health improvements [20].

## 5. Conclusions

Since all studies except one showed some reductions in BMI, 11 of 16 studies reported significant BMI improvements, and all studies showed improvements in at least one other health indicator, a tendency toward the effectiveness of the interventions was demonstrated. No clear patterns regarding the effectiveness or significance were found with respect to study design, intensity, and frequency. As interventions conducting aerobic exercises achieved the highest reductions in BMI, focusing on aerobic energy expenditure could be a promising approach for practitioners to reduce BMI. Nevertheless, there is a need for a better understanding of which types of aerobic exercises are most effective and whether this finding is also evident cross-culturally. However, this systematic review showed that physical-activity-based interventions can be effective and, as university students are prone to physical inactivity and overweight and obesity, policymakers should foster health promotion programs in the tertiary sector in the long term. The university as a setting proves to be ideal as it can take advantage of highly educated staff, facilities, and resources that can facilitate building a healthy lifestyle over the course of peoples’ studies (i.e., long-term interventions for long-term effects). Additionally, due to the rising number of students, a large population from different ethnic and socioeconomic backgrounds can be reached. Given that overweight and obesity and physical inactivity are major public health problems and their prevalence continues to increase, further high-quality, large-sample studies on this topic are needed to gain a comprehensive understanding of the effectiveness of weight loss interventions in overweight and obese university students.

## Figures and Tables

**Table 1:** Assessment of study quality using the Quality Assessment Tool for Quantitative Studies (EPHPP).

Study	Selection Bias	Study Design	Con- Founders	Blinding	Data Collection Methods	Withdrawals and Dropouts	Global Rating
Chen et al. (2021) [37]	2	1	1	3	1	1	2
Dalibalta et al. (2017) [30]	2	1	3	2	1	3	3
DiFrancisco-Donoghueet al. (2018) [31]	2	1	1	2	1	1	1
Gifari et al. (2021) [32]	1	1	3	2	1	1	2
Ha and So (2012) [33]	2	1	1	2	1	1	1
Joseph et al. (2014) [41]	2	2	3	2	1	1	2
Joseph et al. (2016) [42]	2	2	3	2	1	1	2
Lee et al. (2021) [43]	2	2	3	3	1	3	3
Mathunjwa et al. (2013) [44]	2	2	3	2	1	1	2
Moravveji et al. (2019) [38]	2	1	1	2	1	1	1
Pacholek et al. (2021) [36]	2	1	3	2	1	3	3
Roopchand-Martin et al. (2015) [45]	3	2	3	2	1	1	3
Siqiang (2018) [34]	2	1	1	2	1	3	2
Sun et al. (2020) [39]	2	1	1	2	1	1	1
Zhang, W. and Yu, L. (2021) [35]	2	1	1	2	1	3	2
Winters-Stone et al. (2020) [40]	2	1	1	2	1	1	1

Ratings: 1= strong, 2 = moderate, 3 = weak.

## Appendix A

**Table A1:** Study characteristics of the included studies.

Source	Design and Theoretical Framework	Population *	Intervention	Duration and Frequency	Intensity
Chen et al. (2021) [37], China	Randomized Controlled Trial	*N* = 37 [0/37] IG (*n* = 18) CG (*n* = 19)	IG: cycling combined with blood flow restriction training (BFRT) by wearing air pressure belts CG: cycling without BFRT	12 weeks (2× per week, 3 sessions/time with 15 min/session)	IG: low-intensity cycling (40% VO_2max_) with BFRT exercises; 1 min rest between sessions CG: low-intensity cycling (40% VO_2max_) without BFRT exercises; 1 min rest between sessions
Dalibalta et al. (2017) [30], United Arab Emirates	Controlled Clinical Trial	*N* = 46 [40/14] IG (*n* = 14) CG (*n* = 32)	IG (BMI ≥ 25): exercise program (not further defined) CG (BMI ≤ 25): exercise program (not further defined)	8 weeks (3× per week with 60 min/session)	Moderate-high intensity (not further defined)
DiFrancisco-Donoghue et al. (2018) [31], USA	Controlled Clinical Trial	*N* = 113 [60/67] Fitbit-Plus group (*n* = 35) Fitbit-Only group (*n* = 38) CG (*n* = 40)	Fitbit-Plus group: wrist activity trackers, participation in weekly mentored walks/runs, weekly emails (Sunday) offering fitness challenges and feedback on step count Fitbit-Only group: wrist activity trackers and instructions on how to use them CG: no wrist activity trackers	39 weeks Fitbit-Plus group: 10.000 steps daily (30–45 min walking)	Not mentioned
Gifari et al. (2021) [32], Indonesia	Controlled Clinical Trial	*N* = 27 [22/27] PWI (*n* = 9) HIIT (*n* = 9) PWI + HIIT (*n* = 9)	PWI: pre-meal water consumption (PWI); 600 mL before each mealtime with a total water intake of 1.8 L/day HIIT: high-intensity interval training (HIIT) HIIT + PWI: combination of PWI and HIIT	8 weeks (3× per week with 18 min/session)	70–85% of HR-max
Ha and So (2012) [33], Korea	Controlled Clinical Trial	*N* = 16 [16/16] IG (*n* = 7) CG (*n* = 9)	IG: combination of aerobic exercise (treadmill running) and resistance exercise (whole body) CG: maintain normal sedentary activities	12 weeks (3× per week with 80 min/session)	Treadmill running: 60–80% of HRR Resistance training: 3 sets of 10–15 RM
Joseph et al. (2014) [41], USA	Cohort Study (1 group and pre–post design)	*N* = 15 [15/15]	aerobic exercise (1) Walking an indoor track (2) Option between exercise on their own or participate in a cardiovascular-based group exercise (Zumba, kickboxing, aerobics)	12 weeks (4× per week with 30–60 min/session)	Moderate (50–70% of HR)
Joseph et al. (2016) [42], USA	Cohort Study (1 group and pre–post design) Social Cognitive Theory (SCT)	*N* = 25 [25/25]	An Internet-enhanced physical activity program consisting of 1) aerobic exercise and 2) an Internet-based application as a promotion tool (1) Walking an indoor track (2) Option between exercise on their own or participate in a cardiovascular-based group exercise (Zumba, kickboxing, aerobics)	12 weeks (4× per week with 30–60 min/session)	Moderate (50–70% of HR)
Lee et al. (2021) [43], Korea	Cohort Study (1 group and pre–post design)	*N* = 10 [10/not mentioned]	High-intensity circuit training 40 min circuit training: 5 min warm-up, 30 min exercise (full body workout), 5 min cool-down	4 weeks (3× per week with 40 min/session)	Week 1–2: 60–70% HRR (RPE Scale = 13–14) Week 2–4: 65–80% HRR (RPE Scale = 14–18)
Mathunjwa et al. (2013) [44], South Africa	Cohort Study (1 group and pre–post design)	*N* = 60 [60/60]	Tae Bo (body fitness exercises combining the moves of taekwondo, karate, boxing, and hip-hop dancing)	10 weeks (3× per week with 60 min/session)	Week 1–5: moderate (Borg RPE Scale = 11–13, 135 bpm) Week 6–10: high (Borg RPE Scale = 14–16, 150 bpm)
Moravveji et al. (2019) [38], Iran	Randomized Controlled Trial	*N* = 31 [31/31] continuous group (*n* = 10) interval group (n = 12) CG (*n* = 9)	1200 m run (each week increase by 400 m) Continuous group: 1200 m without rest intervals Interval group: 3 × 400 m, interspaced with a walking period of 2 min CG: maintain their behavior, should not participate in training programs	8 weeks (3× per week)	60–75% of maximum HR
Pacholek et al. (2021) [36], Saudi Arabia	Controlled Clinical Trial	*N* = 28 [0/not mentioned] COM (*n* = 14) VOL (*n* = 14)	COM: combination program (aerobic and resistance) VOL: volleyball program (isolated skill exercises, small-sided games and volleyball games)	4 weeks (4× per week with 50 min/session)	Subjective feelings with maximal effort (not further defined)
Roopchand-Martin et al. (2015) [45], Jamaica	Cohort Study (1 group and pre–post design)	*N* = 24 [24/13]	Dance intervention utilizing the XBOX Kinect 360 and Just Dance 4	6 weeks (week 1–2: 5 × per week with 30 min/session; week 3–4: 4 × per week with 45 min/session; week 4–6: 3 × per week with 60 min/session)	Moderate (Borg RPE Scale = 12–14)
Siqiang (2018) [34], China	Controlled Clinical Trial	*N* = 100 [100/100] IG (*n* = 50) CG (*n* = 50)	IG: aerobic exercise divided into two (i.e., rope skipping, swimming) CG: intake of calcium pyruvate (2× per day)	12 weeks (4× per week with 60 min/session, divided into two: half an hour in the morning, half an hour in the afternoon)	Moderate (120–150 bpm)
Sun et al. (2020) [39], China	Randomized Controlled Trial	*N* = 300 [0/300] IG (*n* = 150) CG (*n* = 150)	IG: high-intensity interval training (not further defined) CG: aerobic exercise (not further defined)	12 weeks (5× per week)	IG: 4 min with 85% VO_2max_/HR = 174 bpm, then 2 min with 50% VO_2max_, followed by 5 min relaxation (repeated 5×) CG: 40 min with 60% of VO_2max_/HF 140 = bpm
Zhang, W. and Yu, L. (2021) [35], China	Controlled Clinical Trial	*N* = 60 *** [60/60] CG (*n* = 20) IG (*n* = 20) IG + nutrition ** (*n* = 20)	IG: combination of aerobic exercises and resistance training IG + nutrition: resistance training and peer nutrition health education method CG: maintain their behavior	16 weeks (3× per week with 60 min/session)	Aerobic exercises: 30–80% of max HR, according to stage of the training Resistance training: 65–70% of 1-RM, 3 rounds, repetitions not further defined
Winters-Stone et al. (2020) [40], USA	Randomized Controlled Trial	*N* = 77 [58/not mentioned] IG (*n* = 37) CG (*n* = 40)	IG: whole-body vibration (WBV) training CG: should not change their physical activity or dietary habits	24 weeks (3× per week with 20 min/session)	Week 1–3: 30 Hz Week 4: 35 Hz Week 5: 40 Hz Week 6: 45 Hz Week 7–24: 50 Hz

Sample size contains the number of subjects included in the final analysis. IG = intervention group. CG = control group. HR = heart rate. HRR = heart rate reserve. RM = repetition maximum. RPE = rating of perceived exertion. VO_2max_ = maximum oxygen uptake. bpm = beats per minute. * Population characteristics are divided into the number of female and number of overweight people (number of female/number of overweight). ** The results of this group are not considered in Table A2, because of the inclusion of a nutrition intervention. *** Since no drop-outs were reported, the exact number of participants can only be assumed.

**Table A2:** Individual results of the included studies (change of BMI).

Study	Control Group					Intervention Group(s)				
	Baseline	Post-Intervention	Difference Pre–Post (SD)	Difference Pre–Post (%)	ES/Significance	Baseline	Post-Intervention	Difference Pre–Post (SD or CI)	Difference Pre–Post (%)	ES/Significance
	Mean BMI (SD)	Mean BMI (SD)				Mean BMI (SD)	Mean BMI (SD)			
Chen et al. (2021) [37]	30.30 (1.08)	29.90 (1.44)	−0.40	−1.32	d_av_ = 0.317	30.10 (0.95)	29.0 (1.79)	−1.10	−3.65	d_av_ = 0.803 *
Dalibalta et al. (2017) [30]	21.60 (2.05)	21.10 (2.00)	−0.50	−2.31	d_av_ = 0.247 *	28.70 (3.27)	28.10 (2.79)	−0.60	−2.09	d_av_ = 0.198 *
DiFrancisco-Donoghue et al. (2018) [31]			Men: −0.30 (0.50) Women: −0.20 (1.20)					Fitbit-Plus men: 0.20 (1.70) Fitbit-Plus women: −0.50 (0.50) Fitbit-Only men: −0.10 (2.10) Fitbit-Only women: 0.40 (2.30)		
Gifari et al. (2021) [32]						PWI: 26.6 (3.6) HIIT: 25.9 (2.4) HIIT + PWI: 25.7 (3.4)	PWI: 26.7 (3.6) HIIT: 25.8 (2.4) HIIT + PWI: 25.2 (3.2)	PWI: 0.1 HIIT: −0.1 HIIT + PWI: −0.05	PWI: 0.38 HIIT: −0.38 HIIT + PWI: −1.95	PWI: d_av_ = 0.028 HIIT: d_av_ = 0.042 HIIT + PWI: d_av_ = 0.152 *
Ha and So (2012) [33]	24.18 (1.63)	24.38 (1.66)	0.20	0.83		24.97 (2.73)	24.76 (3.01)	−0.21	−0.84	d_ppc2_ = 0.179
Joseph et al. (2014) [41]						33.27 (6.53)	32.57 (6.62)	−0.70	−2.10	d_av_ = 0.106 *
Joseph et al. (2016) [42]						33.80 (5.70)	32.90 (6.00)	−0.90	−2.66	d_av_ = 0.154
Lee et al. (2021) [43]						25.14 (2.51)	24.24 (2.60)	−0.9 (0.35)	−3.58	d_av_ = 0.352 *
Mathunjwa et al. (2013) [44]						32.26 (5.65)	30.11 (5.46)	−2.15	−6.66	d = 0.2 * (1)
Moravveji et al. (2019) [38] (2)	27.57 (1.62)	28.04 (1.46)	0.47	1.70		Continuous group: 26.77 (1.53) Interval group: 27.57 (1.02)	Continuous group: 26.08 (1.21) Interval group: 26.82 (0.98)	Continuous group: −0.69 Interval group: −0.75	Continuous group: −2.58 Interval group: −2.72	Continous group: d_ppc2_ = 0.704 * (2) Interval group: d_ppc2_ = 0.896 * (2)
Pacholek et al. (2021) [36]						COM: 31.30 (8.61) VOL: 26.50 (7.71)	COM: 31.30 (8.36) VOL: 26.20 (7.45)	COM: 0 VOL: −0.3	COM: 0 VOL: −1.13	COM: d_av_ = 0 VOL: d_av_ = 0.04
Roopchand-Martin et al. (2015) [45]						30.51 (5.18)	30.31 (5.39)	−0.2	−0.66	d_av_ = 0.038
Siqiang (2018) [34]	26.80 (3.40)	24.10 (2.90)	−2.70	−10.07	d_av_ = 0.857 *	25.50 (2.70)	21.60 (3.10)	−3.90	−15.29	d_av_ = 1.345 *
Sun et al. (2020) [39]	30.54 (3.86)	28.45 (2.76)	−2.09	−6.84	d_av_ = 0.631 *	30.85 (3.79)	28.17 (2.89)	−2.68	−8.69	d_av_ = 0.802 *
Zhang, W. and Yu, L. (2021) [35] (3)	22.54 (0.71)	22.68 (1.11)	0.14	0.62		22.06 (0.98)	21.64 (0.85)	−0.42	−1.90	d_ppc2_ = 0.641 *
Winters-Stone et al. (2020) [40]	28.27 (26.48; 30.07)	Not available (4)	0.64 (0.31; 0.96)	2.26		Total: 28.24 (23.87; 32.60) High adherence: 27.02 (22.34; 31.71) (5)	Not available (4)	Total: 0.22 (−0.59; 1.03) High adherence: −0.01 (−1.63; 0.86)	Total: 0.78 High adherence: −0.04	Total: *p* = 0.094 High adherence: *p* = 0.026 *

Determined level of significance *p* = 0.05 (* *p* < 0.05). SD = standard deviation. ES = effect size (d_av_ sensu Cumming, 2012; d_ppc2_ sensu Morris, 2008). (1) Mathunjwa et al. (2013) reported effect sizes on their own. (2) Inconsistently reported significance in Table 1 and text. (3) Nutrition group is excluded. (4) Winters-Stone et al. (2020) reported only a 6-month change. (5) Participants with high adherence (≥80% of prescribed sessions).
